# Outcomes of a Pragmatic Randomized Clinical Trial of an Advance Care Planning Program in Nursing Homes

**DOI:** 10.1093/geroni/igaf122.155

**Published:** 2025-12-31

**Authors:** Susan Hickman, Timothy Stump, Abigail Evans, Wanzhu Tu, Kathleen Unroe

**Affiliations:** Indiana University, Indianapolis, Indiana, United States; Indiana University Bloomington, Bloomington, Indiana, United States; Regenstrief Institute, Indianapolis, Indiana, United States; Indiana University, Indianapolis, Indiana, United States; Indiana University School of Medicine, Indianapolis, Indiana, United States

## Abstract

Advance care planning (ACP) helps prepare residents and family caregivers for communication and decision-making in advance of a crisis and helps ensure that preferences are known, documented, and honored. This is particularly important in persons with Alzheimer’s disease or related dementias (ADRD), given known complications that necessitate decisions about hospitalization, resuscitation, feeding, and infection management. We conducted an embedded pragmatic cluster randomized clinical trial to evaluate the implementation of the ACP Specialist Program in 132 nursing homes between September 2021 – August 2022. Existing nursing home staff members were trained to enhance care through improved ACP procedures, standardized online staff education on ACP, and systematic ACP facilitation. Outcomes were assessed using Medicare claims and Minimum Data Set 3.0 assessments, consistent with the pragmatic design. Across the intervention (n = 64) and control (n = 64) buildings, 37% (14,498/39,114) of residents were identified as having ADRD per documented diagnosis of Alzheimer’s or dementia and/or a Cognitive Function Scale score > 2 (moderate or severe cognitive impairment). In the intervention buildings, 77 ACP Specialists engaged in ACP conversations for 21% (N = 1,470) of residents with ADRD. In a mixed effects Poisson regression analysis adjusted for covariates, there were no significant differences between intervention and control sites in our primary outcome of hospital transfers (admissions and emergency department visits) over the 12-month study period (Incident Rate Ratio for intervention versus control = 0.91 (0.79, 1.05), p = ns). Implementation challenges and variable rates of implementation across intervention buildings likely contributed to the non-significant outcome.

